# Evaluation of Focal Liver Reaction after Proton Beam Therapy for Hepatocellular Carcinoma Examined Using Gd-EOB-DTPA Enhanced Hepatic Magnetic Resonance Imaging

**DOI:** 10.1371/journal.pone.0167155

**Published:** 2016-12-01

**Authors:** Shigeyuki Takamatsu, Kazutaka Yamamoto, Yoshikazu Maeda, Mariko Kawamura, Satoshi Shibata, Yoshitaka Sato, Kazuki Terashima, Yasuhiro Shimizu, Yuji Tameshige, Makoto Sasaki, Satoko Asahi, Tamaki Kondou, Satoshi Kobayashi, Osamu Matsui, Toshifumi Gabata

**Affiliations:** 1 Department of Radiotherapy, Kanazawa University Hospital, Ishikawa, Japan; 2 Proton Therapy Center, Fukui Prefectural Hospital, Fukui, Japan; 3 Department of Radiology, Nagoya University Graduate School of Medicine, Aichi, Japan; 4 Department of Radiology, Hyogo Ion Beam Medical Center, Hyogo, Japan; 5 Department of Radiology, University of Fukui, Fukui, Japan; 6 Department of Diagnostic and Therapeutic Radiology, Kanazawa Medical University, Ishikawa, Japan; 7 Department of Radiology, Kanazawa University, Ishikawa, Japan; Northwestern University Feinberg School of Medicine, UNITED STATES

## Abstract

**Background:**

Proton beam therapy (PBT) achieves good local control for hepatocellular carcinoma (HCC), and toxicity tends to be lower than for photon radiotherapy. Focal liver parenchymal damage in radiotherapy is described as the focal liver reaction (FLR); the threshold doses (TDs) for FLR in the background liver have been analyzed in stereotactic ablative body radiotherapy and brachytherapy. To develop a safer approach for PBT, both TD and liver volume changes are considered clinically important in predicting the extent of damage before treatment, and subsequently in reducing background liver damage. We investigated appearance time, TDs and volume changes regarding FLR after PBT for HCC.

**Material and Methods:**

Patients who were treated using PBT and were followed up using gadolinium ethoxybenzyl diethylenetriamine pentaacetic acid-enhanced magnetic resonance imaging (Gd-EOB-DTPA MRI) after PBT were enrolled. Sixty-eight lesions in 58 patients were eligible for analysis. MRI was acquired at the end of treatment, and at 1, 2, 3 and 6 months after PBT. We defined the FLR as a clearly depicted hypointense area on the hepatobiliary phase of Gd-EOB-DTPA MRI, and we monitored TDs and volume changes in the FLR area and the residual liver outside of the FLR area.

**Results:**

FLR was depicted in all lesions at 3 months after PBT. In FLR expressed as the 2-Gy equivalent dose (α/β = 3 Gy), TDs did not differ significantly (27.0±6.4 CGE [10 fractions [Fr] vs. 30.5±7.3 CGE [20 Fr]). There were also no correlations between the TDs and clinical factors, and no significant differences between Child-Pugh A and B scores. The volume of the FLR area decreased and the residual liver volume increased, particularly during the initial 3 months.

**Conclusion:**

This study established the FLR dose for liver with HCC, which might be useful in the prediction of remnant liver volume for PBT.

## Introduction

Recently, highly conformal radiotherapy, used as stereotactic ablative body radiotherapy (SABR), has been delivered safely and effectively for hepatocellular carcinoma (HCC) [1]. Furthermore, particle beam therapies such as proton beam therapy (PBT) and carbon ion therapy have been reported to achieve good local control regarding HCC [2,3]. In a systematic review and meta-analysis, toxicity tended to be lower using such particle beam radiotherapies relative to photon radiotherapy [4]. However, damage to the liver parenchyma in PBT has not been well evaluated.

The focal liver parenchymal effect after SABR appears as a low-density area on computed tomography (CT) or a hypointense area during the hepatobiliary phase of gadolinium ethoxybenzyl diethylenetriamine pentaacetic acid-enhanced magnetic resonance imaging (Gd-EOB-DTPA MRI). This effect is described as the focal liver reaction (FLR) [5–7], and is a useful marker for predicting liver parenchymal damage in radiotherapy. For this purpose, using the hepatobiliary phase of Gd-EOB-DTPA MRI, the threshold dose (TD) for the background liver has been analyzed in patients with metastatic liver tumors and HCC associated with chronic liver disease in SABR and brachytherapy [7,8]. In PBT, Yuan et al. were the first to report on FLR and MRI-based dosimetric proton end-of-range verification for the liver [9], but they did not examine TD in their analysis.

Previous reports concerning the TD in photon therapy analyzed using Gd-EOB-DTPA MRI have shown shrinkage in the volume of the irradiated liver [8,10,11]. Thus, the volume of the FLR in the liver would also decrease after PBT. Consequently, we hypothesized that for analyzing TD in relation to FLR, the expected volumetric change of the irradiated liver parenchyma should be taken into account. Additionally, Imada et al. have reported that compensatory enlargement in the non-irradiated liver after carbon ion therapy contributes to an improved prognosis [12]. Taken together, to develop a safer approach to PBT, both the FLR TD and volume change in the liver irradiated at doses exceeding the TD or in non-irradiated liver are considered to be clinically important in predicting the extent of the damage before treatment, and subsequently reducing background liver damage.

In the present study, we attempted to investigate the appearance time, TDs and volume changes in the FLR using Gd-EOB-DTPA-enhanced MRI after PBT for hepatocellular carcinoma.

## Materials and Methods

### Patients and clinical examination

**Fig 1:**
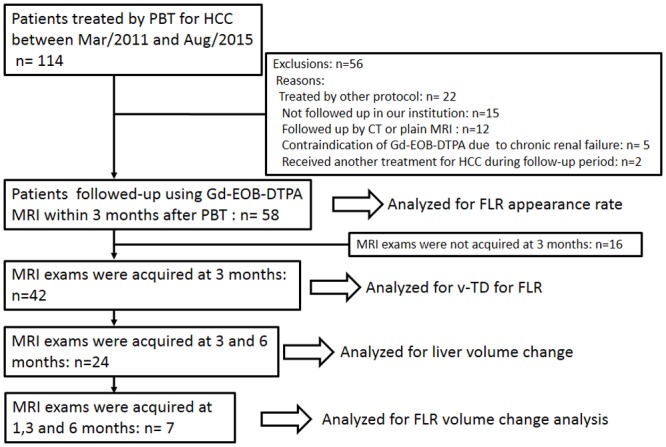
Flow diagram for the patient selection process. Abbreviations; PBT: Proton beam therapy; HCC: Hepatocellular carcinoma; Gd-EOB-DTPA MRI: Gadolinium ethoxybenzyl diethylenetriamine pentaacetic acid-enhanced magnetic resonance imaging; CT: Computed tomography; FLR: Focal liver reaction; v-TD: Visual-threshold dose; dFLR: destined FLR area.

This retrospective analysis of the data was approved by the institutional review board of our institution, and written informed consent was obtained from each patient. Between March 2011 and August 2015, patients who were treated using PBT for HCC at total doses of 66 cobalt Gy equivalent (CGE)/10 fractions (Fr) or 76 CGE/20 Fr, and followed up using Gd-EOB-DTPA MRI within 3 months after PBT were enrolled. Patients were not eligible for this study if they had the following characteristics: HCC treated using PBT in combination with transcatheter arterial chemoembolization (TACE); HCC <2 cm distant from the digestive tract; or they did not receive follow-up MRI in our institution; or they were treated repeatedly as a result of HCC recurrence or new HCC lesions within 6 months after the first PBT. Fifty-eight patients were considered eligible for analysis (Table 1; Fig 1).

**Table 1:** Patient characteristics.

Characteristics	n
Patients	58
Gender, male/female	35/23
Median age (range), years	73 (56–90)
PS 0/1/2	49/8/1
Lesions	68
Median tumor size (range), mm	28 (5–105)
Median PTV volume (range), cm3	89.2 (18.2–879.7)
Median liver volume (range), cm3	1150.8 (642.7–2117.1)
Child Pugh A/B	48/10
Prior treatment TACE/RFA/PEIT/surgery	20/10/5/2
Tumor thrombus PV/HV/bile duct	6/2/1
Chronic hepatitis HCV/HBV/alcoholic/others	35/7/5/10
Total dose 66 CGE/76 CGE	32/26

**Abbreviations**; PS: performance status; PTV: Planning target volume; TACE: Transcatheter arterial chemoembolization; RFA: radiofrequency ablation; PEIT: percutaneous ethanol injection therapy; PV: portal vein; HV: hepatic vein; HCV: hepatitis C virus; HBV: hepatitis B virus; CGE: Cobalt Gy equivalent.

The diagnosis of HCC was made clinically by means of early nodular staining regarding the arterial dominant phase and “wash out” in the equilibrium phase of dynamic CT and/or MRI, and using serum levels of tumor markers (alfa-fetoprotein [AFP] and des-gamma-carboxy prothrombin [PIVKA-II]) [13].

The initial workup for these patients generally included a thorough medical history and physical examination. All patients underwent blood tests, including complete blood cell counts, liver and renal function tests and determination of electrolytes, hepatitis B and C virus titers, AFP and PIVKA-II. Abdominal enhanced CT and MRI were performed.

### Pretreatment imaging and PBT planning

The patients were positioned in the supine position and immobilized using a custom-induced vacuum-lock bag and a low-temperature thermoplastic body cast (Esform: Engineering System Co., Nagano, Japan). Respiratory synchronized 4-dimensional CT (4D-CT) (Aquilion LB TSX-201A: Toshiba Medical Systems Co., Tochigi, Japan) was obtained during the expiratory phase under the following conditions: 120 kv; 300 mA; and 2.0-mm-thick consecutive slices for treatment planning of PBT. Respiratory gating was controlled by abdominal wall motion with the laser sensor of a respiratory gating system (AZ-733V: Anzai Medical Co., Tokyo, Japan).

MRI examination was performed after CT examination for planning. The magnetic resonance apparatus used was a 1.5 Tesla system (Signa HDx 1.5T Optima Edition: General Electric Healthcare, Waukesha, USA). The patient received a dose of 0.1 ml/kg of Gd-EOB-DTPA (Primovist: Bayer Schering Pharma, Berlin, Germany) injected at 1.5 ml/sec. The hepatobiliary phase was acquired at 15 min after post-contrast T1-weighted acquisition, performed with the 3-dimensional spoiled gradient-recalled acquisition in steady state. Liver acquisition was performed with volume acceleration, extended volume with fat-saturation (LAVA-XV; repetition time/echo time 4.3/2.0 ms; flip angle = 15 degrees; field of view 32×32–40×40 cm; matrix 320×192×88 or 96; interpolated to 512× 512; acquisition time 16–23 sec), and the slice thickness was 4 mm with a slice gap of 2 mm; there was cessation of respiration during the expiratory phase for approximately 20 sec.

**Fig 2:**
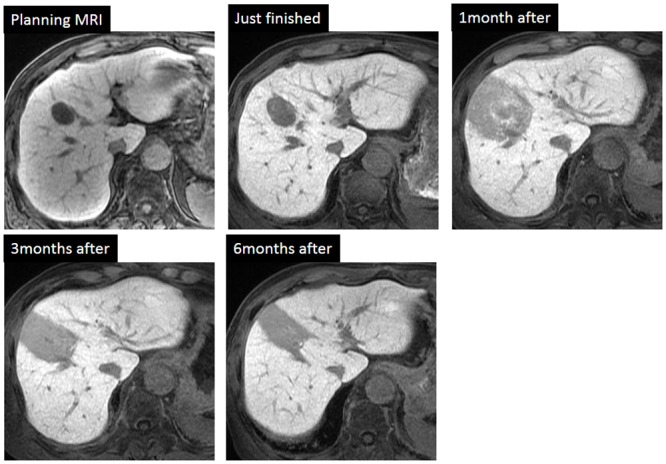
Gd-EOB-DTPA MRI scans showing changes in the irradiated HCC and the background liver after PBT. On the follow-up Gd-EOB-DTPA MRI scan, FLR did not appear 1 month later in this case. The contours of the FLR were made manually on MR images at 3 or 6 months after PBT. The dFLR contour (50% isodose line in this case) is defined as the area irradiated at doses exceeding the TD at planning MRI and gradually shrinks to the FLR area at 3 or 6 months after PBT. HCC, white arrow head; dFLR contour, yellow; FLR contour, green line. Abbreviations; Gd-EOB-DTPA MRI: Gadolinium ethoxybenzyl diethylenetriamine pentaacetic acid-enhanced magnetic resonance imaging; HCC: Hepatocellular carcinoma; PBT: Proton beam therapy; FLR: Focal liver reaction; destined FLR area: dFLR; TD: Threshold dose.

For PBT planning, a 3-dimensional treatment planning system (Xio-N: Elekta, Stockholm, Sweden; Mitsubishi Electric Corporation, Kobe, Japan) was used. Diagnostic CT or planning MRIs were fused with planning CT images acquired by 4D-CT at the expiratory phase for target delineation with rigid registration. Gross tumor volume (GTV) was defined by MRI using dynamic contrast-enhanced images and with Gd-EOB-DTPA during the hepatobiliary phase. The clinical target volume included a 5-mm radial expansion of the GTV to target possible microscopic disease extension. To compensate for respiratory movement, ITV margins calculated using respiratory movement analysis with planning 4D-CT and the planning target volume (PTV) was expanded by 5 mm in all directions with an additional 5- to 7-mm margin in the craniocaudal direction. Some patients with daughter lesions were irradiated once using PBT as the combined PTV. The proton beam treatment plan mainly involved two or three ports, so the border of the treatment area resembled a straight line; furthermore, the irradiated area was planned so that it included the tumor blood drainage area to avoid local recurrence. Therefore, the FLR area resembled the defects area after surgical segmental resection (Fig 2).

A total dose of 76 CGE in 20 Fr was selected for tumors within 2 cm of the porta hepatis, and 66 CGE in 10 Fr for tumors located in peripheral segments of the liver, using an irradiation schedule of 5 Fr per week. The radiation dose was prescribed in CGE using a relative biological effectiveness value of 1.1, based on our preclinical experiments. The total dose at the isocenter was prescribed to cover 95% of the PTV. The PBT system used a synchrotron and a passive scattering method (Proton Beam System: Mitsubishi Electric Corporation, Kobe, Japan). Daily irradiation was performed via more than two ports (with the exception of the plan using a one port beam). A respiratory gating system (AZ-733V) was used to synchronize treatment in the expiratory phase.

### Gd-EOB-DTPA MRI analysis for FLR appearance time and threshold doses

Gd-EOB-DTPA MRI analysis to determine the appearance time of FLR and TD analysis. The analysis was performed by two radiation oncologists with over 10 years of experience by discussion and consensus (S.T. and K.Y.), using commercially available software (MIM Maestro: MIM Vista Corp, Cleveland, OH, USA).

TD analysis was carried out using the visual decision method and volume change analysis using the volume data by means of the visual decision method. We defined the FLR as a clearly depicted hyposignal intensity area relative to the surrounding liver parenchyma in the hepatobiliary phase of Gd-EOB-DTPA MRI after PBT (Fig 2). To determine the appearance time of FLR, the rate of visualization of FLR among the MRI examinations at each time point was analyzed. In this study, the FLR TD was analyzed using MR images obtained at 3 months after PBT.

**Fig 3:**
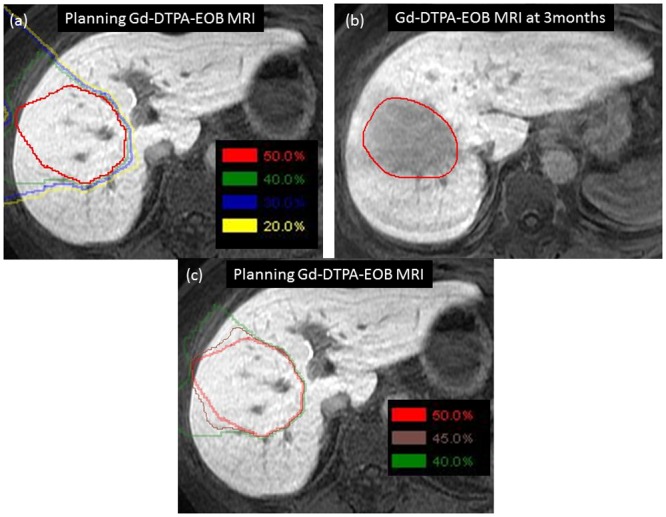
Method for defining the TD for FLR using hepatobiliary phase Gd-EOB-DTPA MRI at 3 months after PBT. The isodose lines were transferred onto each Gd-EOB-DTPA MRI. (a) The isodose lines are displayed as 50%, 40%, 30% and 20% lines. These dose lines were compared with the FLR contour on the planning MRI. (b) The medial dose line (45% line; brown) between the selected two doses (40% and 50% lines) was added. Finally, using these three isodose lines, the dose distribution contours that were most similar to the FLR contour were defined. In this case, TD was defined by the 45% dose line as 29.7 CGE. FLR contour, red (on the left side); 50% isodose line, red; 45% isodose line, brown; 40% isodose line, green; 30% isodose line, blue; 20% isodose line, yellow. Abbreviations; TD: Threshold dose; FLR: Focal liver reaction; Gd-EOB-DTPA MRI: Gadolinium ethoxybenzyl diethylenetriamine pentaacetic acid-enhanced magnetic resonance imaging; PBT: Proton beam therapy; CGE: Cobalt Gy equivalent.

Fig 3, S1 Fig shows the visual decision method of analysis of the TD for FLR in the hepatobiliary phase of Gd-EOB-DTPA MRI. The TD defined by this method was termed the visual-TD (v-TD). In defining the TDs, we compared the dose contours on the planning MRI with the FLR contour on the subsequent MRI. The dose contours made from the isodose lines of the prescribed dose were created on the planning CT and the dose contours were transferred onto each MRI using rigid registration.

We estimated the isodose lines for the v-TD using the positional relationship information, while visually comparing the positions of the dose lines with the FLR contours. Anatomically, we referred to the relevant blood vessels, hepatic lobules, ligaments and cysts. In relation to the v-TD definition method, isodose lines are displayed in every 10% line (from 10% to 100% dose lines). These dose lines were compared with the FLR contour (red) on the planning MRI (Fig 3a and S1 Fig). Two isodose lines resembling the v-TD were selected in every 10% line. In Fig 3b, the isodose lines are displayed as 50% (red), 40% (green), 30% (blue) and 20% (yellow) lines. In this case, similar lines were the 40% and 50% lines. The medial dose line (45% line; brown) between the selected two doses was added (Fig 3b). Finally using these three isodose lines, the dose distribution contours that were most similar to the FLR contour were defined. The dose contour that was most similar to the FLR contour for each 5% line was defined as the v-TD for each patient by the radiation oncologists by consensus. If the selection of the v-TD contours using the three isodose lines was difficult, the lowest one was adopted. In this case, the v-TD was defined by the 45% dose line as 29.7 CGE (Fig 3b). Next the v-TD was calculated from the total dose for each protocol. To determine intra-observer reproducibility of v-TD measurement, the analyses were repeated again at 1 month intervals, and the mean values of each data set were used.

To compare the v-TD of 66 CGE and 76 CGE, we used equivalent doses in 2 CGE fractions (EQD2), which takes into account the total dose and the dose per fraction. EQD2 was calculated using the following equation: EQD2 = D × ([d + α/β)/(2 + α/β]), as derived from the linear-quadratic (LQ) model, where D is the total dose, d is the fractionation dose, α is the linear component of cell killing, β is the quadratic component of cell killing and the α/β ratio is the effect on the normal tissue of a dose of 3 Gy [14].

**Fig 4:**
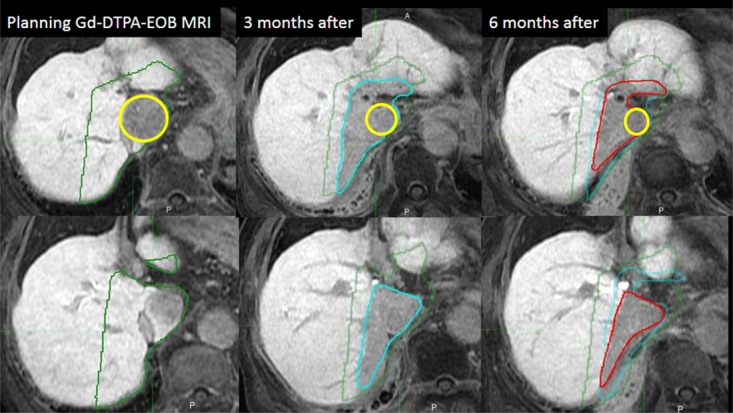
Representative examples of the volume changes of the dFLR or FLR area at planning and on follow-up MRI (upper stage, cranial section; lower stage, caudal section). The dFLR area including the HCC gradually shrank after PBT. dFLR area at planning (252 cm^3^), green; FLR area at 3 months (107 cm^3^), sky blue; FLR area at 6 months (72 cm^3^), red; HCC, yellow. Abbreviations; dFLR: destined focal liver reaction area; FLR: Focal liver reaction; HCC: Hepatocellular carcinoma; PBT: Proton beam therapy; v-TD: visual-threshold dose.

In volume analysis, the contours of the whole liver were set to include the hepatic vein, hepatic portion of the inferior vena cava and the second branch of the portal vein. On the follow-up MRI scan, the contours of the FLR area were made. Two radiation oncologists (S.T. and K.Y.) delineated the whole liver and the FLR on MRI, and the volumes were calculated. Based on the v-TD defined by MRI at 3 months after PBT, we hypothesized the irradiated liver area receiving a dose greater than the v-TD isodose lines at planning MRI as the destined FLR area (dFLR). The volume of the dFLR was calculated and compared with the FLR volume on follow-up MRI. To determine intra-observer reproducibility when measuring FLR volume, FLR volumes were delineated and measured twice at 1 month intervals, and the mean values of each set of measurements were used. The liver volume outside of the dFLR or FLR area (residual liver) was also calculated. In addition, the change in each volume was compared at each MRI examination interval (Fig 4).

We hypothesized that the FLR volume after PBT was gradually decreased in a manner resembling exponential decay with a decay constant of -1/T, and finally converged after a long time lapse. Based on this hypothesis, the volume was expressed by the following equation:

$$\text{V}\left(\text{t}\right)\;=\Delta \text{V}e^{\text{-t/T}}+{\text{ V}}_{\text{R}},$$ (1)

where t is the time (months) after PBT, T is the mean lifetime of the FLR volume change, and ΔV and V_R_ are the volume change and the residual volume at the eternal time, respectively. The parameters ΔV, V_R_ and T were evaluated by fitting the relative FLR volume at 1 month and 6 months for all possible lesions by means of least squares method; the relative FLR volume was obtained each month and divided by the volume at 3 months, and thus the volume at 3 months was equal to 1. Then the V(0) data obtained by the extrapolation of the function at month zero were defined as the calculated FLR (cFLR). To evaluate the reasonability of the equation using exponential decay, V(0) data were also obtained using a linear function for comparison. cFLR data were compared with the dFLR volume calculated using the v-TD to evaluate the appropriateness of our visual anatomical method.

### Statistical methods

In defining the TD method concerning the isodose lines, the data values were acquired in multiples of 5%; we defined the TD as a categorical variable. To characterize the reproducibility of TD dose line definition, weighted kappa statistics were used [15].

Correlations among TDs expressed in EQD2 and clinical factors (gender, age, chronic liver disease with viral infection, alcoholic liver, Child–Pugh (CP) score, CP class, prior treatment, prior treatment in field and whole liver volume), planning factors (dose per Fr, tumor size, PTV volume) and volume change factors at follow-up Gd-EOB-DTPA MRI (volume change of the whole liver, the FLR area and outside of the FLR area) were assessed using Pearson correlation coefficients and multiple regression analysis.

To determine intra-observer reproducibility when measuring the FLR volume, FLR volume analysis was carried out using Pearson’s correlation coefficient and the Bland–Altman method [16]. Comparisons between two independent groups were analyzed using the Mann–Whitney U test, and related data were analyzed using the Wilcoxon signed-rank test to identify all differences. A p-value of <0.05 was considered statistically significant. Statistical analyses were performed using IBM SPSS 20.0 (IBM SPSS, Chicago, IL, USA) software.

## Results

Gd-EOB-DTPA MRI was performed after the completion of PBT at intervals of 1, 2, 3 and 6 months at the patient’s convenience. One-hundred twenty two follow-up MRI images obtained at the end of treatment and at 1, 2, 3 and 6 months after PBT were analyzed, namely 13, 34, 9, 42 and 24 MRI images at each time point, respectively.

In the analysis of FLR appearance, the patients examined within 3 months after PBT were enrolled; 58 patients were analyzed using 123 follow-up MRI images. The median time of appearance of FLR was 3 (range, 0–3) months. The rate of appearance of FLR at each time point was as follows: 8% (1/13) at the end of treatment; 47% (16/34) at 1 month; 67% (6/9) at 2 months; 100% (42/42) at 3 months; and 100% (24/24) at 6 months after treatment (Figs 1 and 2). At 3 months after treatment all lesions had developed FLR. Consequently, the following analysis of the TD regarding the FLR was used at 3 months after PBT.

**Fig 5:**
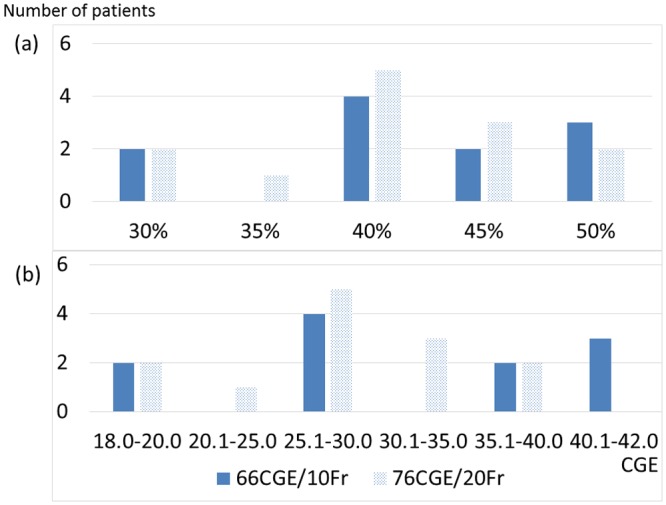
Distribution of the prescribed dose (%) (a) and the threshold doses calculated using EQD2 (b). Abbreviations; EQD2: Equivalent doses in 2 CGE fractions; CGE: Cobalt Gy equivalent; Fr: Fractions.

In the TD analysis, the patients examined at 3 months after PBT were enrolled; 42 patients were analyzed (Fig 1). The v-TD for the prescribed dose had a median value of 40% (range, 30–50%; 19.8–38.0 CGE) and EQD2 had a median value of 27.5±7.0 (range, 18.9–41.6) CGE. The difference between median relative TD and absolute TD was the result of the two different levels of prescribed doses (Table 2; Fig 5). There were also no correlations between the TDs and clinical factors with multiple regression analysis, and no statistical differences between CP-A and CP-B (Table 2).

**Table 2:** Threshold doses for focal liver reaction.

	66 CGE/10 Fr	76 CGE/20 Fr	CP-A	CP-B
TD for FLR (CGE) (mean±SD)	25.7±4.5	29.5±4.7	NA	NA
TD of EQD2 for FLR (CGE) (mean±SD)	27.0±6.4	30.5±7.3	29.1±7.1	27.0±6.6

Abbreviations; TD: Threshold dose; FLR: Focal liver reaction; EQD2: Equivalent doses in 2 CGE fractions; CGE: Cobalt Gy equivalent; Fr: Fractions; CP: Child Pugh.

**Fig 6:**
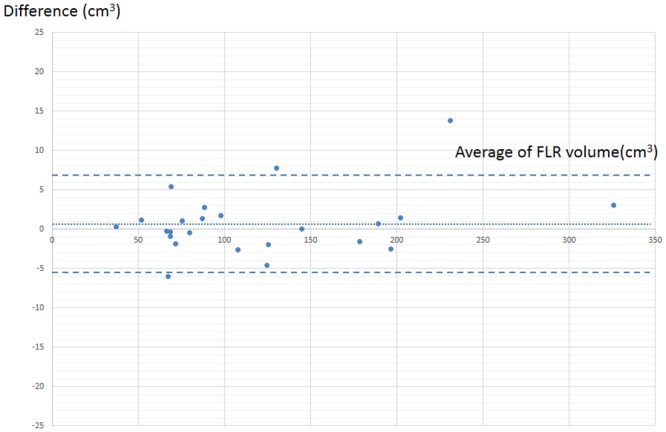
Bland–Altman plot for the method comparison study (n = 24). The small dashed line denotes bias (mean difference) and the large dashed lines denote 95% limits of agreement (2 SD of difference). Abbreviations; SD, standard deviation; FLR: Focal liver reaction.

In volume change analysis, the patients examined at both 3 and 6 months after PBT were enrolled; 24 patients were analyzed (Table 3). In FLR volume analysis, we found a good reproducibility between intra-observer error for the FLR volume (mean difference, 2.2% [range, −6.0–13.8] cm^3^; Pearson's correlation coefficient, R(2) = 0.99]). The Bland–Altman plot involving the method comparison study is shown in Fig 6.

**Table 3:** 

		Pretreatment (mean±SD) n = 24	3 months after (mean±SD) n = 24	6 months after (mean±SD) n = 24
**dFLR or FLR area**	**Volume (cm3)**	**220.5±98.3**	**121.0±69.5**	**88.1±58.0**
	**Proportion of FLR at 3 months (%)**	**197.4±50.6**	**100**	**71.9±11.9**
**Residual liver**	**Volume (cm3)**	**924.5±257.3**	**1006.2±269.4**	**1005.5±242.0**
	**Proportion of FLR at 3 months (%)**	**92.3±9.1**	**100**	**100.6±7.4**
**Total liver**	**Volume (cm3)**	**1145.0±284.7**	**1127.2±285.9**	**1093.6±255.7**
	**Proportion of FLR at 3 months (%)**	**102.1±7.7**	**100**	**97.7±7.3**

Abbreviations; dFLR: destined focal liver reaction; FLR: Focal liver reaction; SD, standard deviation. Changes in volume values and the rate of variability in comparison with the values at planning are detailed. Values are given as the mean ± standard deviation.

The bias (mean difference) ± twice the precision (95% limits of agreement: 2 SD of difference) equaled −0.7±6.2 cm^3^; this demonstrated the good reproducibility of the FLR visual analysis.

**Fig 7:**
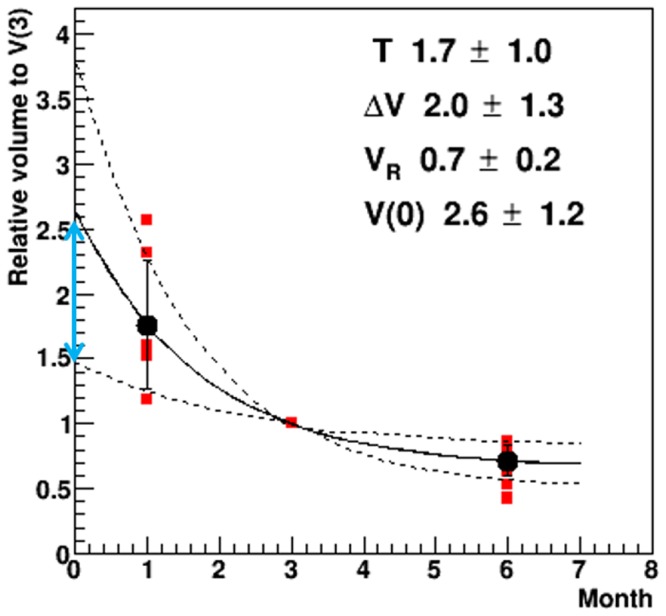
Volume change in the FLR as a function of time (months) after PBT. The square plots (red) show the FLR volume relative to the volume at 3 months (V(1), V(3) and V(6) data), and the circles (black) with error bars denote the mean and the standard deviations of the volume. The solid and dashed curve show the mean value and one standard deviation the calculated result of Eq (1), respectively. The two direction arrow shows the range of dFLR calculated using the v-TD (refer to Table 2). The relative dFLR volume calculated from v-TD for 30% and 50% of the prescription dose were estimated to be 2.4±0.3 and 1.7±0.4, respectively, and were found to be similar (within the standard deviation) to the V(0) (cFLR) of Eq (1) rather than the value of a linear function. Abbreviations; FLR: Focal liver reaction; PBT: Proton beam therapy; Eq: equation; dFLR: destined focal liver reaction area; v-TD: visual-threshold dose; cFLR: calculated FLR.

In the Eq (1) analysis, the patients examined at 1, 3 and 6 months after PBT were enrolled; seven cases that exhibited FLR changes were analyzed (Fig 1). Fig 7 shows the volume change in the dFLR and cFLR as a function of time (months) after PBT; all of the FLR volumes evaluated at the subsequent MRI were calculated relative to the volume at 3 months. The curve in the figure shows the calculated result of Eq (1), and the value of T and ΔV was estimated to be 1.7±1.0 months and 2.0±1.3 months, respectively, where the errors corresponds to one standard deviation. The relatively large errors were caused by the large variation in the volume at one month among seven cases; This results in that the value of V(0) was estimated to be 2.6+-1.2. In contrast the value of V(0) evaluated using a linear function was 1.3±0.2 and had a smaller value than the V(1) data.

The relative dFLR volume among 7 cases calculated from v-TD for 30% and 50% of prescription dose were estimated to be 2.4±0.3 and 1.7+-0.4, respectively, and were found to be similar (within the standard deviation) to the V(0) of Eq (1) rather than the value of a linear function.

In addition, to evaluate the appropriateness of the exponential decay function, we found that individual data sets at 1-, 3-, 6-, 9- and 12-month time points were well fitted by the equation, but not by a linear function. The results are shown in S2 and S3 Figs, where 25 points for five cases are presented and the thick solid curves have been fitted using Eq (1). Thus, we consider that it may be reasonable to use Eq (1) for the evaluation of V(0) rather than a linear function. This might indicate that the volume change differs from that calculated using a linear function. The volume of the FLR area decreased and the residual liver increased over a period of 6 months, especially during the initial 3 months (Table 3).

## Discussion

Recently, the possible utility of Gd-EOB-DTPA MRI for estimating liver functional reserve has been reported [17]. One of the major histopathological changes caused by liver irradiation is sinusoidal obstruction syndrome (SOS) (formerly known as hepatic veno-occlusive disease) [17,18]. A recent study indicated that the primary damage site in SOS is the centrilobular (zone 3 of the liver parenchyma) sinusoidal endothelial cell.

Gd-EOB-DTPA is incorporated into hepatocytes mainly by organic anion transporting polypeptide (OATP). It has been clarified that there is a highly significant correlation between Gd-EOB-DTPA uptake and OATP1B3 expression in HCC cells and/or normal hepatocytes, and also between the grade of OATP1B3 expression and the enhancement ratio (signal intensity) during the hepatobiliary phase of Gd-EOB-DTPA MRI [19,20]. OATP1B3 is known to be predominantly expressed in zone 3 hepatocytes in normal liver parenchyma [21]. We believe that these factors are responsible for the well-defined visualization of the FLR during the hepatobiliary phase of Gd-EOB-DTPA MRI; thus, we were able to evaluate the damage to the liver caused by PBT irradiation. However, Richter et al. reported a hypothesis concerning the potential molecular mechanism responsible for radiation-induced changes in hepatocyte-specific Gd-EOB-DTPA [22]; a number of other factors may exist in relation to this issue [23].

Considering the volume change, the volume of the FLR area decreased over a period of 6 months after therapy; this change was more rapid during the initial 3 months. The volume for the residual liver increased, especially during the initial 3 months. According to a study on adult living donor liver transplantation [24], rapid initial regeneration of the remnant donor liver occurred in a similar manner to that reported in the current study. This finding might be of use as a reference concerning the time interval for repetitive PBT for an additional HCC lesion.

There are some data regarding TD that have been reported in previous studies using various protocols (Table 4). The median appearance time for FLR in the present study was 3 (range, 0–3) months. This appearance time may be dose-dependent and influenced by the different time periods used for treatment delivery. Single high-dose irradiation might cause damage earlier than fractionated irradiation using the same biologically effective dose treatment. Our findings and those of other reports are consistent with this theory. However, there are differences in the timing of the appearance of FLR in each patient. The timing did not show a uniform trend; for example, cases with lower or higher TD did not have the tendency to exhibit the earlier appearance of FLR. Timing may have been affected by background liver conditions (e.g., fibrosis, hemodynamics and liver function).

**Table 4:** The diversity of threshold doses for the focal liver reaction.

Author	n	Treatment modality	TD	Fr	EQD2			Back ground liver	Image analysis modality	Consideration of volume change	Timing of analysis
					α/β = 2	α/β = 3	α/β = 8				
Seidensticker [8]	23	HDR brachytherapy	9.4	1	26.8	23.3	16.4	normal liver	Gd-EOB-DTPA MRI	-	-
Rühl [11]	20	HDR brachytherapy	9.1–9.6	1	25.3–27.8	22.0–24.2	15.6–16.9	normal liver	Gd-BOPTA MRI	-	6 weeks
Herfarth [6]	36	SABR	13.7	1	53.7	45.8	29.7	most of the normal liver	CE-CT	-	1.8 months
Sanuki [7]	35	SABR	30	5	60	54	42	chronic hepatitis:CP-A	Gd-EOB-DTPA MRI	GTV only	3 months
Sanuki [7]	10	SABR	25	5	43.8	40	32.5	chronic hepatitis:CP-B	Gd-EOB-DTPA MRI	GTV only	3 months
Present study	11	PBT	27	10	31.7	30.8	28.9	chronic hepatitis	Gd-EOB-DTPA MRI	GTV and back ground	3 months
Present study	13	PBT	30.5	20	26.9	27.6	29.1	chronic hepatitis	Gd-EOB-DTPA MRI	GTV and back ground	3 months

Abbreviations; HDR, high dose rate; SABR: Stereotactic ablative body radiotherapy; PBT: Proton beam therapy; TD: Threshold dose; Fr: Fractions; EQD2: Equivalent doses in 2 CGE fractions; CP: Child Pugh; Gd-EOB-DTPA MRI: Gadolinium ethoxybenzyl diethylenetriamine pentaacetic acid-enhanced magnetic resonance imaging; Gd-BOPTA, gadobenate dimeglumine; CE-CT: contrast enhanced computed tomography.

In EQD2 using the LQ model, although previous analyses have incorporated various α/β ratios [7, 8, 11, 25], the most suitable α/β ratio for normal liver or liver with chronic disease remains unknown. A range of TD values have been reported in these studies, likely attributable to the influence of different TD determination methods with or without considering the volume change. The TD expressed in EQD2 is approximately 30 CGE, and these data are not conflicting considering the classical liver tolerance dose; the mean liver dose for radiation-induced liver disease involving whole liver irradiation was 30 Gy [26].

A dose calculation method involving definition of the TD should be adaptable to a volume change in the FLR and residual liver after irradiation. There is a risk that the TD for FLR can be overestimated in comparisons of the planning dose lines at planning with the FLR demarcation lines after irradiation in the fusion images from planning CT or MRI scans and follow-up MRI images. Because the FLR area shrinks over time and the smaller volume of the shrunken FLR was compared with the planning dose lines or dose-volume histogram, the calculated TD can be higher than the actual TD. Our technique entails simple visual judgments regarding TD definition without use of rigid or non-rigid fusion techniques; thus, it contains ambiguous factors, but it can flexibly take into consideration anatomical change in the relevant blood vessels, hepatic lobules and ligaments; consequently, it can adapt to volume change in the GTV and liver parenchyma irradiated to a higher dose than the TD. Seidensticker et al. reported on a possible time dependence regarding the TD as a result of volume change in the FLR, resulting in a difference in TD at each time point after irradiation [8]. They did not consider the volume reduction in the irradiated liver at doses higher than the TD. Other studies involving TD after radiotherapy did not consider the FLR volume change after irradiation in the TD calculation method [6,7,11]. Considering the nature of the FLR volume change after irradiation, there were some uncertainties with respect to the method used to define the TD. However, in our comparison of the dFLR volume from the v-TD and V(0) data from the equation there were no inconsistencies. This indicates that our v-TD definition method is appropriate.

Furthermore, it was difficult to compare 1) the TD for normal liver with that for liver with chronic disease, and 2) the results of single-fraction high-dose irradiation with our results using the LQ model. Uncertainties also stemmed from the following: differences in the irradiated volume of the target area; the use of combination treatment with TACE; the daily reproducibility of planning conditions; and the differences in the characteristics of photon and proton beams. However, these issues could not be resolved in the current study, which to our knowledge, is the first report on FLR TDs after PBT for HCC accompanied by chronic liver disease. In our results, the TDs did not correlate with any factors and did not significantly differ between patients with CP-A and CP-B. This may be attributable in part to the smaller number of patients in the CP-B group than in the CP-A group in our study. Otherwise, the liver in both CP-A and CP-B patients might be similarly vulnerable to PBT.

The present study had several limitations. The FLR and background liver volumes change with time after PBT. The irradiated area shrinks and the background liver enlarges, so the trend in volume change in each region differs in the same liver. Therefore, using the TD definition method, the application to non-rigid registration is difficult and we used rigid registration following the estimation method, taking into consideration each volume change. Accordingly, our TD definition method entailed a potential error. In addition, we did not acquire MRI images at all monthly follow-up periods for all patients; therefore, the patient sample size was limited at some time points, especially at 2 months after MRI.

However, in relation to PBT for HCC, the TD calculated in the current study and the volume analysis data could promote greater safety and less invasive radiation exposure to background liver. Further study is necessary to clarify the present analysis involving PBT, with more frequent (e.g., weekly) MRI follow-ups after PBT.

## Conclusions

FLR was detectable in all cases at 3 months after PBT in Gd-EOB-DTPA MRI scans. The volume of the FLR area decreased and the residual liver volume increased over a 6-month period after treatment, especially during the initial 3 months. Using an α/β ratio of 3, FLR doses expressed in EQD2 were nearly 30 CGE in the liver of patients with HCC. These data might be useful in the prediction of the remnant liver volume.

## Supporting Information

S1 Fig Method for defining the TD for FLR using hepatobiliary phase Gd-EOB-DTPA MRI at 3 months after PBT. (a) The isodose lines were transferred onto each Gd-EOB-DTPA MRI. (b) The isodose lines are displayed as 60%, 50% and 40% lines. These dose lines were compared with the FLR contour on the planning MRI. (c) The medial dose line (45% line; brown) between the selected two doses (40% and 50% lines) was added. Finally, using these three isodose lines, the dose distribution contours that were most similar to the FLR contour were defined. In this case, TD was defined by the 45% dose line as 29.7 CGE. FLR contour, red; 60% isodose line, pink; 50% isodose line, red; 45% isodose line, brown; 40% isodose line, green. Abbreviation; TD: Threshold dose; FLR: Focal liver reaction; Gd-EOB-DTPA MRI: Gadolinium ethoxybenzyl diethylenetriamine pentaacetic acid-enhanced magnetic resonance imaging; PBT: Proton beam therapy; CGE: Cobalt Gy equivalent.
(TIF)

S2 Fig Volume change in the FLR as a function of time (months) after PBT. All data points at 1-, 3-, 6-, 9- and 12-month time points in five patients are presented; they were fitted using Eq (1). The square plots (red) show the FLR volume relative to the volume at 3 months (V(1), V(3), V(6), V(9) and V(12) data), and the circles (black) with error bars denote the mean and the standard deviations of the volume. The solid and dashed curve shows the mean value and one standard deviation, respectively of the data calculated using Eq (1). The two direction arrow show the range of dFLR calculated using the v-TD (refer to Table 2). Abbreviations; FLR: Focal liver reaction; PBT: Proton beam therapy; dFLR: destined focal liver reaction.
(TIF)

S3 Fig Volume change in the FLR as a function of time (months) after PBT. The square plots (red) show the FLR volume relative to the volume at 3 months (V(1), V(3), V(6),V(9) and V(12) data) in each of the five cases. Abbreviations; FLR: Focal liver reaction; PBT: Proton beam therapy.
(TIF)

S1 File Dataset for clinical data, liver volume, and FLR doses. (XLSX)

## Abbreviations

4D-CT: 4-dimensional CT
AFP: Alfa-fetoprotein
CGE: Cobalt Gy equivalent
CP: Child Pugh
CT: Computed tomography
cFLR: calculated FLR
dFLR: destined FLR area
EQD2: Equivalent doses in 2 CGE fractions
Eq: equation
FLR: Focal liver reaction
Fr: Fractions
Gd-BOPTA: gadobenate dimeglumine
Gd-EOB-DTPA MRI: Gadolinium ethoxybenzyl diethylenetriamine pentaacetic acid-enhanced magnetic resonance imaging
GTV: Gross tumor volume
HBV: hepatitis B virus
HCC: Hepatocellular carcinoma
HCV: hepatitis C virus
HDR: high dose rate
HV: hepatic vein
LQ model: linear-quadratic model
mo: month(s)
OATP: organic anion transporting polypeptide
PBT: Proton beam therapy
PEIT: percutaneous ethanol injection therapy
PIVKA-II: Des-gamma-carboxy prothrombin
PS: performance status
PTV: Planning target volume
PV: portal vein
RFA: radiofrequency ablation
SABR: Stereotactic ablative body radiotherapy
SD: standard deviation
SOS: Sinusoidal obstruction syndrome
TACE: Transcatheter arterial chemoembolization
TD: Threshold dose
TDs: Threshold doses
tTD: tentative threshold dose
