# Supplementary figures and images for: Evaluation of Focal Liver Reaction after Proton Beam Therapy for Hepatocellular Carcinoma Examined Using Gd-EOB-DTPA Enhanced Hepatic Magnetic Resonance Imaging
Source: PLoS One. 2016 Dec 1;11(12):e0167155. doi: 10.1371/journal.pone.0167155 (PMC5132228; doi:10.1371/journal.pone.0167155)

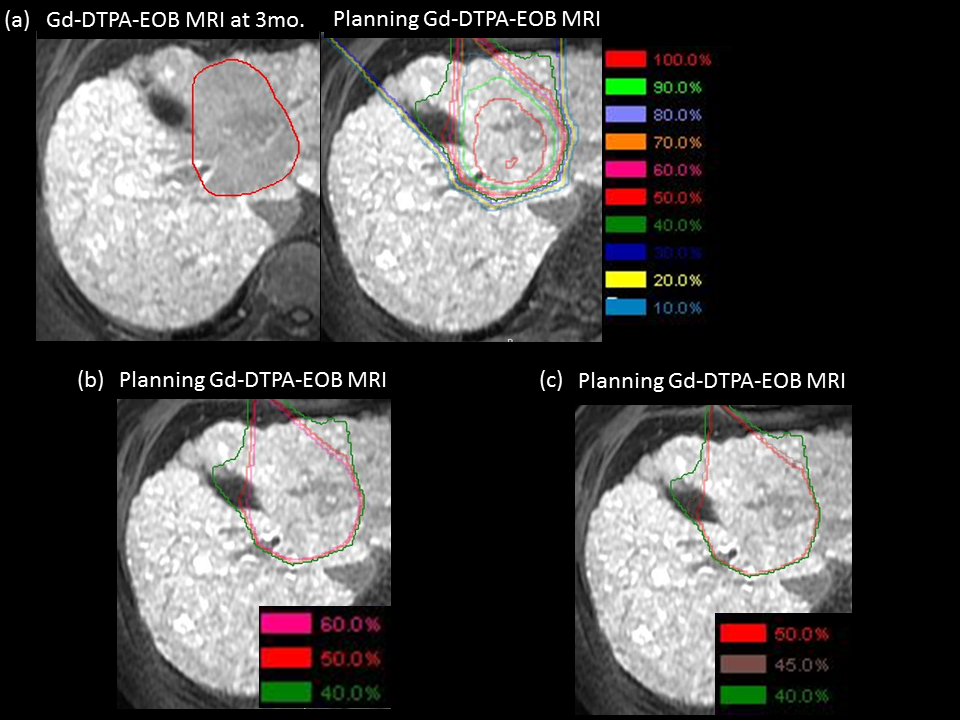

Supplement: S1 Fig — (a) The isodose lines were transferred onto each Gd-EOB-DTPA MRI. (b) The isodose lines are displayed as 60%, 50% and 40% lines. These dose lines were compared with the FLR contour on the planning MRI. (c) The medial dose line (45% line; brown) between the selected two doses (40% and 50% lines) was added. Finally, using these three isodose lines, the dose distribution contours that were most similar to the FLR contour were defined. In this case, TD was defined by the 45% dose line as 29.7 CGE. FLR contour, red; 60% isodose line, pink; 50% isodose line, red; 45% isodose line, brown; 40% isodose line, green. Abbreviation; TD: Threshold dose; FLR: Focal liver reaction; Gd-EOB-DTPA MRI: Gadolinium ethoxybenzyl diethylenetriamine pentaacetic acid-enhanced magnetic resonance imaging; PBT: Proton beam therapy; CGE: Cobalt Gy equivalent. (TIF) [file pone.0167155.s001.tif]

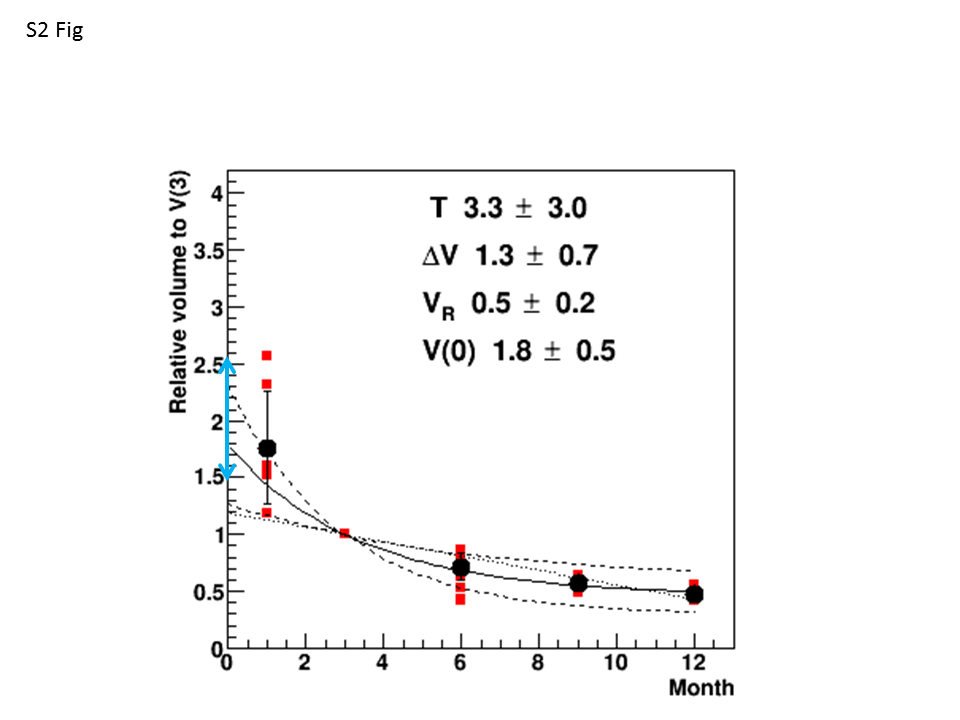

Supplement: S2 Fig — All data points at 1-, 3-, 6-, 9- and 12-month time points in five patients are presented; they were fitted using Eq (1). The square plots (red) show the FLR volume relative to the volume at 3 months (V(1), V(3), V(6), V(9) and V(12) data), and the circles (black) with error bars denote the mean and the standard deviations of the volume. The solid and dashed curve shows the mean value and one standard deviation, respectively of the data calculated using Eq (1). The two direction arrow show the range of dFLR calculated using the v-TD (refer to Table 2). Abbreviations; FLR: Focal liver reaction; PBT: Proton beam therapy; dFLR: destined focal liver reaction. (TIF) [file pone.0167155.s002.tif]

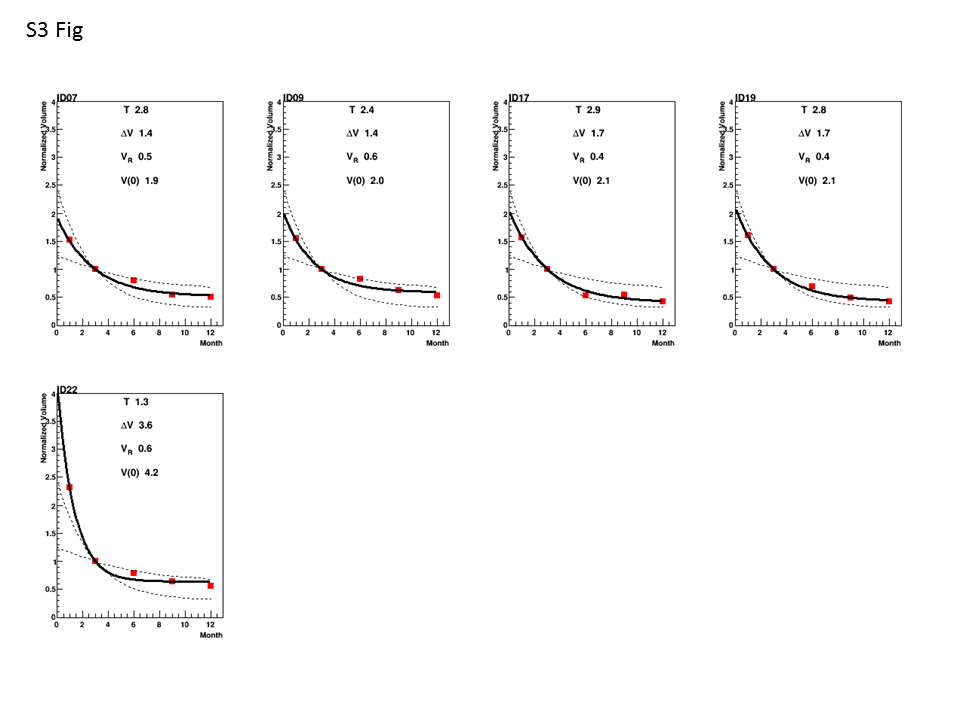

Supplement: S3 Fig — The square plots (red) show the FLR volume relative to the volume at 3 months (V(1), V(3), V(6),V(9) and V(12) data) in each of the five cases. Abbreviations; FLR: Focal liver reaction; PBT: Proton beam therapy. (TIF) [file pone.0167155.s003.tif]
